# Age-related shifts in gut microbiota contribute to cognitive decline in aged rats

**DOI:** 10.18632/aging.103093

**Published:** 2020-05-01

**Authors:** Yanli Li, Li Ning, Yiru Yin, Rui Wang, Zhiyong Zhang, Lijun Hao, Bin Wang, Xin Zhao, Xiaorong Yang, Litian Yin, Shufen Wu, Dawei Guo, Ce Zhang

**Affiliations:** 1 Key Laboratory of Cellular Physiology, Ministry of Education, Department of Physiology, Shanxi Medical University, Taiyuan 030001, Shanxi, P.R. China; 2 Department of Neurology, First Hospital of Shanxi Medical University, Taiyuan 030001, Shanxi, P.R. China; 3 College of Information and Computer, Taiyuan University of Technology, Taiyuan 030024, Shanxi, P.R. China

**Keywords:** aging-related cognitive decline, gut microbiota, fecal microbiota transplantation, operant-based delayed matching to position task, resting state fMRI

## Abstract

Cognitive function declines during the aging process, meanwhile, gut microbiota of the elderly changed significantly. Although previous studies have reported the effect of gut microbiota on learning and memory, all the reports were based on various artificial interventions to change the gut microbiota without involvement of aging biological characteristics. Here, we investigated the effect of aged gut microbiota on cognitive function by using fecal microbiota transplantation (FMT) from aged to young rats. Results showed that FMT impaired cognitive behavior in young recipient rats; decreased the regional homogeneity in medial prefrontal cortex and hippocampus; changed synaptic structures and decreased dendritic spines; reduced expression of brain-derived neurotrophic factor (BDNF), N-methyl-D-aspartate receptor NR1 subunit, and synaptophysin; increased expression of advanced glycation end products (AGEs) and receptor for AGEs (RAGE). All these behavioral, brain structural and functional alterations induced by FMT reflected cognitive decline. In addition, FMT increased levels of pro-inflammatory cytokines and oxidative stress in young rats, indicating that inflammation and oxidative stress may underlie gut-related cognitive decline in aging. This study provides direct evidence for the contribution of gut microbiota to the cognitive decline during normal aging and suggests that restoring microbiota homeostasis in the elderly may improve cognitive function.

## INTRODUCTION

Aging is characterized by a progressive functional decline that is inevitable in the life process [1]. During aging, the brain undergoes a myriad of alterations, including biological, psychological, neuroanatomical, and neurophysiological changes, which are closely tied to the decline in cognitive functions. However, the age-related cognitive decline is extremely complex, and the mechanisms remain largely unclear.

The human gastrointestinal tract harbors a complex and dynamic population of some 10^14^ microorganisms, referred to as gut microbiota [2]. The gut microbiota is very important for the development and homeostasis of the body; it regulates intestinal motility and gastrointestinal barrier [3], host energy metabolism and mitochondrial function [4], as well as immune responses and the central nervous system [2]. In adulthood, the microbiota reaches a relative equilibrium, and does not significantly change under stable environmental and health conditions. Generally, the phyla *Bacteroidetes* and *Firmicutes* dominate the intestine for adulthood. However, with an increasing age, the gut microbiota undergoes a profound remodeling. Claesson et al. have shown that the gut microbiota of the elderly is substantially different from the younger adults [5], and correlates with frailty measured by functional independence measure (FIM) [6]. However, given our current inability to delineate the most significant effector mechanisms involved in the host-microbiota interactions over a lifetime, it is difficult to tease apart causality from correlation.

Although some animal studies indicated that the gut microbiota affects learning and memory [7], these reports were based on special animal models, such as germ-free (GF) mice [8], or on various artificial interventions that change the gut microbiota, such as pathogenic bacterial infection, probiotics [8], and antibiotics [9]. Since the aging process and aging biological characteristics were not considered in these studies, they were not able to uncover the association between gut microbiota and cognitive function under normal aging process.

Given these findings, we hypothesized that alterations in the gut microbiota contribute to cognitive decline in aging. In this study, we transplanted the gut microbiota from aged rats to young rats by using the fecal microbiota transplantation (FMT) technique, to observe whether the reshaped gut microbiota can cause a shift in cognitive behavior, brain structure, and functions in the young recipient rats. To our knowledge, this is the first study that investigates the effect of gut microbiota on cognitive decline in normal aging process.

## RESULTS

### Cognition changes in aged rats

**Figure 1:**
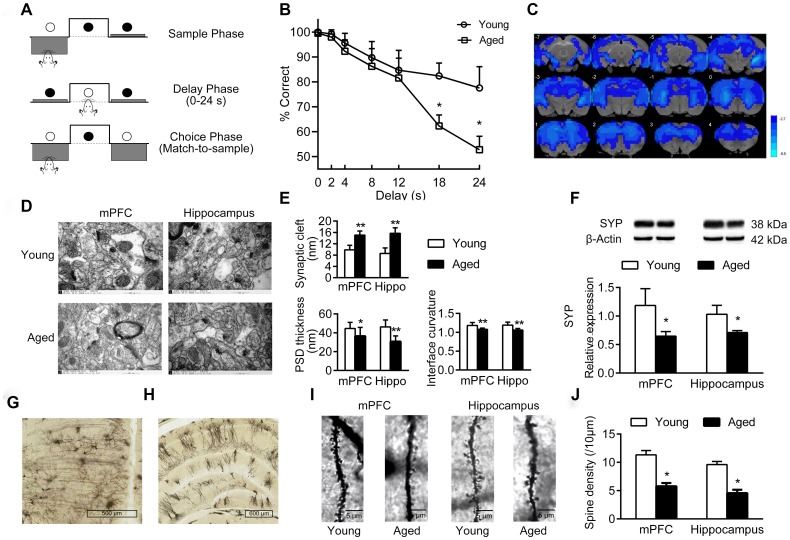
**Cognition changes in aged rats.** (**A**) The DMTP procedure, consisting of sample phase, delay phase and choice phase. ○ illumination of the stimulus light or panel light, ● extinguished stimulus light or panel light. (**B**) Cognitive performance of young and aged rats analyzed by DMTP; n = 12. (**C**) Images of brain slices showing regions with lower ReHo in aged rats compared with young rats; n = 20. (voxel level < 0.005, cluster level < 0.05 GRF corrected, and clusters > 50 voxels). Blue denotes lower ReHo; the color bars indicated the T values between groups. (**D**) The synaptic structures of mPFC and hippocampus in young and aged rats by transmission electron microscopy (× 60000). n = 3. (**E**) Histograms of synaptic structure parameters. n = 3. (**F**) Expression of synaptophysin in mPFC and hippocampus by western blot. SYP: synaptophysin. n = 3. (**G**–**J**) Golgi staining performed on mPFC and hippocampus of young and aged rats (n = 3). Representative Golgi staining images of the mPFC (**G**) and hippocampus (**H**) demonstrate impregnation of neurons. (**I**) Representative images of dendritic spines. (**J**) Quantification of dendritic spine densities in mPFC and hippocampus. Error bars represent the SEM; * *P* < 0.05, ** *P* < 0.01 compared to young rats.

The cognitive functions of young and aged rats were analyzed by operant-based delayed matching to position (DMTP) task (Figure 1A). The correct rate of lever-pressing at different delays is shown in Figure 1B. At the shortest delay, both young and aged rats performed well. As delays increased, the accuracy decreased in both young and aged rats, especially at 18 s and 24 s delay, the accuracy decreased significantly (main effect of delay: *P* < 0.05). Notably, compared with young rats, aged rats performed even worse at longer delays of 18 s and 24 s, and the accuracy was significantly lower than in the young rats (*P* < 0.05). When the delay was 24 s, the accuracy in aged rats was close to 50%, possibly a probability event (Figure 1B).

Using resting-state functional magnetic resonance (rs-fMRI) and regional homogeneity (ReHo) analytical method, we identified the brain regions showing differences in spontaneous blood oxygenation level dependent (BOLD) signal, representing neuronal activities in young and aged rats. Compared with young rats, aged rats had lower ReHo values in almost the entire brain; this is consistent with the structural and functional alterations in normal aging brain (Figure 1C).

In addition, there were significant changes of synaptic structures in medial prefrontal cortex (mPFC) and hippocampus in aged rats, including widened synaptic cleft, thinned post-synaptic density (PSD), and decreased curvature of synaptic interface (Figure 1D–1E). Aged rats had a decreased expression of synaptophysin in mPFC and hippocampus compared to young rats (*P* < 0.05, Figure 1F). Furthermore, the density of dendritic spines in mPFC and hippocampus decreased significantly in aged rats (Figure 1G–1J).

**Figure 2:**
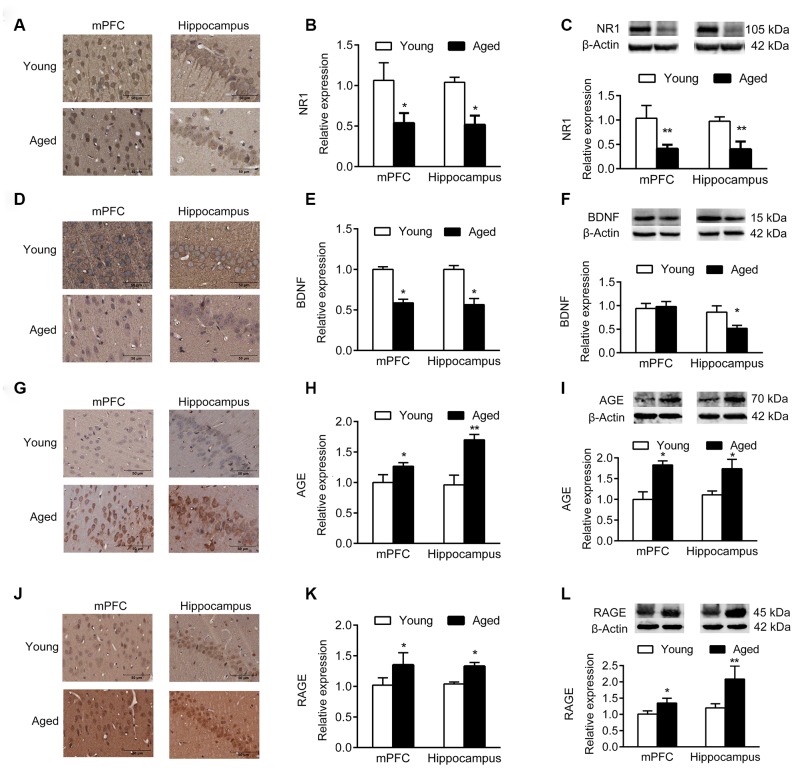
**Expression of molecular markers in young and aged rats. n = 3.** (**A**–**C**) Expression of NR1 in mPFC and hippocampus by IHC (**A**, **B**), and western blotting (**C**). (**D**–**F**) Expression of BDNF in mPFC and hippocampus by IHC (**D**, **E**), and western blotting (**F**). (**G**–**I**) Expression of AGE in mPFC and hippocampus by IHC (**G**, **H**), and western blotting (**I**). (**J**–**L**) Expression of RAGE in mPFC and hippocampus by IHC (**J**–**K**), and western blotting (**L**). Error bars represent the SEM. * *P* < 0.05, ** *P* < 0.01.

Immunohistochemistry (IHC) and western blotting analyses demonstrated that the expression of NR1, the essential subunit of N-methyl-D-aspartate (NMDA) receptor, decreased significantly in aged mPFC and hippocampus compared with young rats (Figure 2A–2C). In addition, the expression of brain derived neurotrophic factor (BDNF) in mPFC and hippocampus of aged rats decreased compared with young rats (Figure 2D–2F), while the expression of advanced glycation end products (AGEs) and receptor for AGEs (RAGE) increased (Figure 2G–2L).

**Figure 3:**
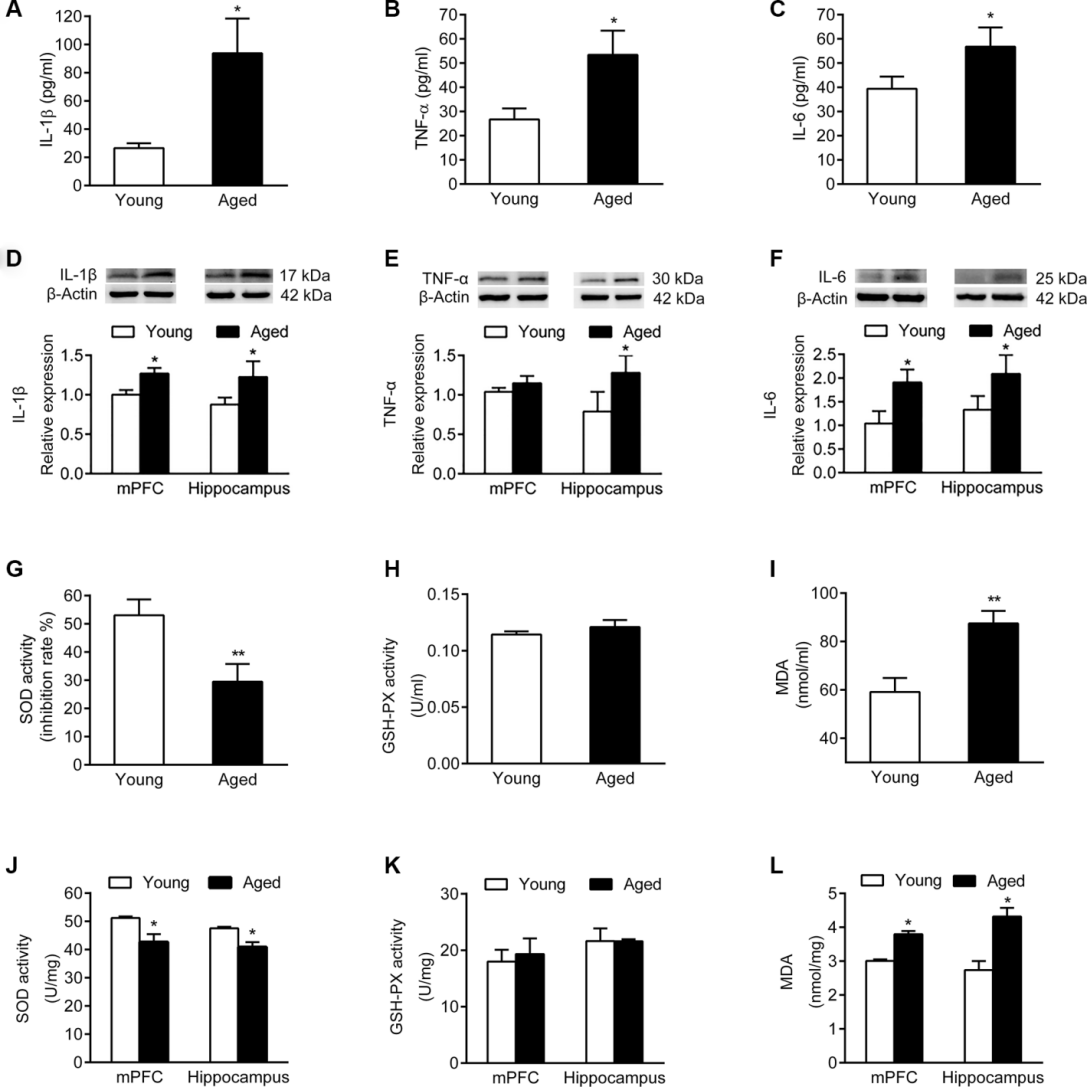
**Inflammatory cytokines and oxidative stress markers in serum and brain of young and aged rats. n = 6.** Serum levels of IL-1β (**A**), TNF-α (**B**), and IL-6 (**C**) in young and aged rats. Expression of IL-1β (**D**), TNF-α (**E**), and IL-6 (**F**) in mPFC and hippocampus in young and aged rats. SOD activity (**G**), GSH-PX activity (**H**), and MDA content (**I**) in serum of young and aged rats. SOD activity (**J**), GSH-PX activity (**K**), and MDA content (**L**) in mPFC and hippocampus of young and aged rats. Error bars represent the SEM. * *P* < 0.05, ** *P* < 0.01.

Analysis of inflammatory cytokines in serum and brain tissues showed that the serum levels of IL-1β, TNF-α and IL-6 were increased in aged rats compared with young rats (Figure 3A–3C). Similarly, the expression of IL-1β, TNF-α and IL-6 was higher in mPFC and hippocampus of aged rats than in young rats (Figure 3D–3F).

We then examined activity of the antioxidant enzymes superoxide dismutase (SOD) and glutathione peroxidase (GSH-PX), as well as the expression of malondialdehyde (MDA). In serum, the SOD activity of aged rats declined, while the MDA expression increased; the activity of GSH-PX did not change compared to young rats (Figure 3G–3I). Similarly, both in mPFC and hippocampus of aged rats, the SOD activity declined, while the MDA level increased compared with young rats; the activity of GSH-PX did not change (Figure 3J–3L).

### Gut microbiota varies in young and aged rats

**Figure 4:**
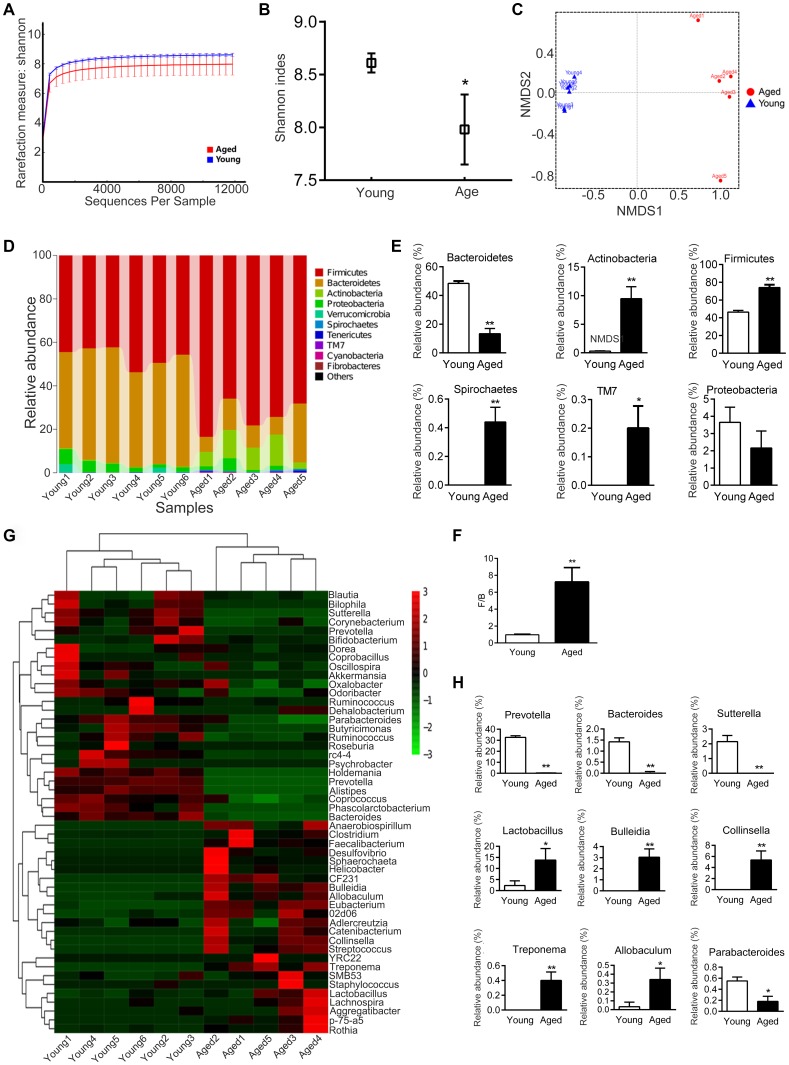
**Gut microbiota varies between young and aged rats. n = 5.** (**A**) Rarefaction curve. (**B**) Shannon diversity index; the higher the Shannon index, the higher the diversity of the community. (**C**) Weighted UniFrac Nonmetric Multidimensional Scaling (NMDS); the distance between two points indicates the similarity of microbial structure. (**D**) Relative abundance of bacteria at phylum in each sample; each column represents one sample. (**E**) Phyla with significant differences between young and aged rats. (**F**) Ratio of *Firmicutes*/*Bacteroidetes* (F/B). (**G**) Heatmap of community composition at genus by cluster analysis. The illustration in the top right corner shows the difference in the expression of bacteria from -3 (green) to +3 times (red). Compared with young rats, red represented an increase in number and green represented a decrease. (**H**) Genera with significant differences between young and aged rats; * *P* < 0.05, ** *P* < 0.01

Analysis of the composition of gut microbiota in young and aged rats showed that the diversity of microbiota was significantly lower in aged rats than in young rats (*P* < 0.05, Figure 4A, 4B). Nonmetric Multidimensional Scaling (NMDS) analysis showed that the microbial communities of aged rats were significantly different from young rats (*P* < 0.05, Figure 4C); there was no overlap between the microbiota of young and aged rats.

We then analyzed the composition of microbiota communities at various taxonomic levels. At the phylum level, *Bacteroidetes* and *Firmicute* were the main bacteria in the feces of rats (Figure 4D). The relative abundance of *Bacteroidetes* was lower in aged rats than in young rats, while the relative abundance of *Firmicutes* was higher (Figure 4E). The ratio of *Firmicutes* / *Bacteroidetes* (F/B) increased about 7.4 fold with aging, from 0.97 ± 0.07 to 7.22 ± 1.69 (*P* < 0.01, Figure 4F). In addition, the relative abundance of *Actinobacteria*, *Spirochaetes* and *TM7* increased in aged rats compared to young rats (Figure 4E). There was no statistical difference in *Proteobacteria* between the young and aged rats (Figure 4E).

At the genus level, the microbiota composition in aged rats significantly differed from the young rats (Figure 4G). The relative abundance of *Prevotella*, *Bacteroides*, *Sutterella* and *Parabacteroides* decreased in aged rats, whereas *Bulleidia*, *Collinsella*, *Lactobacillus*, *Treponema* and *Allobaculum* increased in aged rats (Figure 4H).

### Gut microbiota of young recipient rats is reshaped by fecal microbiota transplantation (FMT)

To analyze the effect of gut microbiota on cognitive decline in aged rats, we treated young rats with aged microbiota from aged rats by FMT. Within 2 months after transplantation, some characteristics of the microbiota in the young recipient rats (3 months) changed to characteristics similar for aged (24 months) rats, including diversity and dominant bacteria.

**Figure 5:**
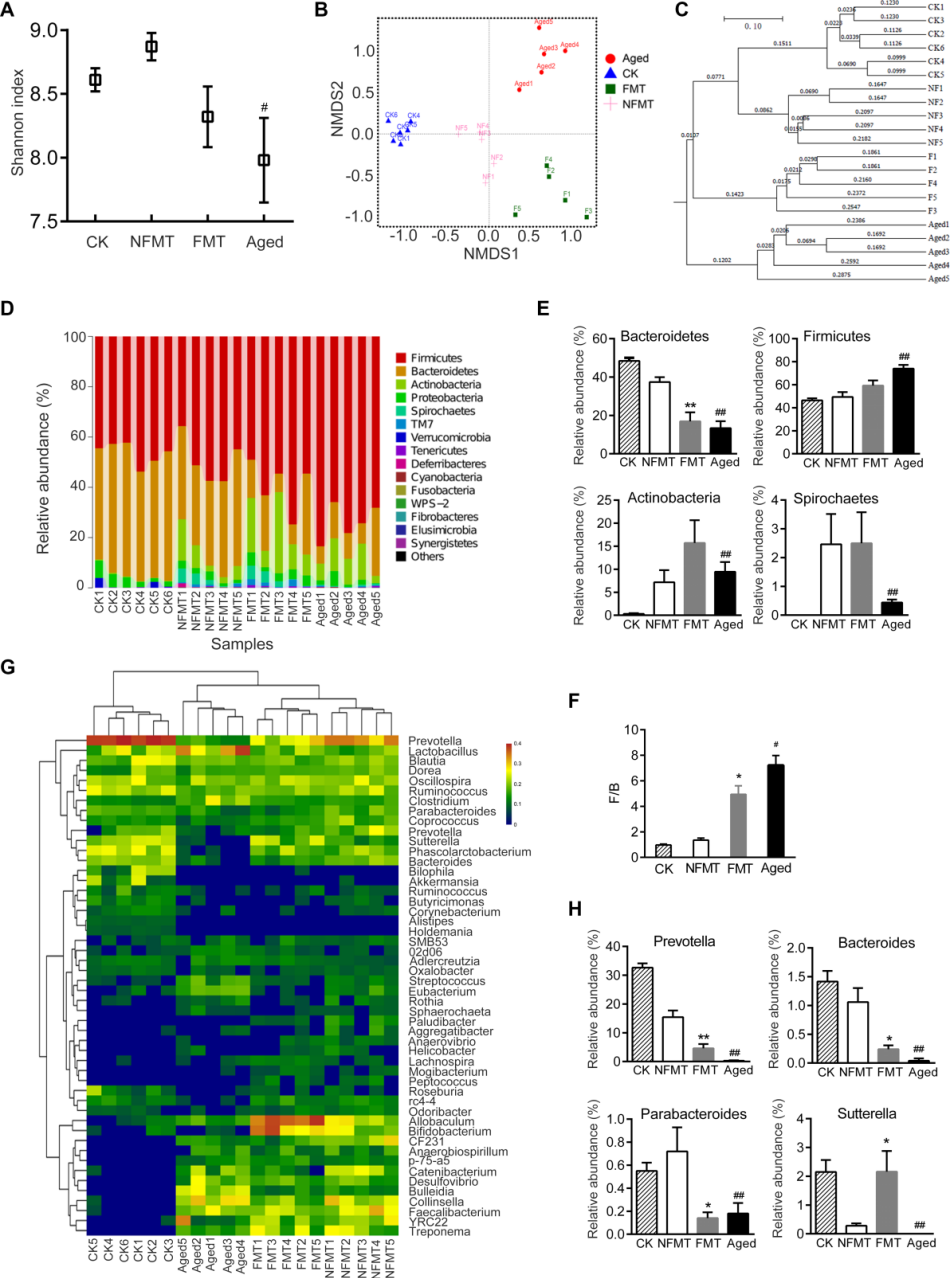
**Gut microbiota of young recipient rats is reshaped by FMT. n = 5.** (**A**) Shannon index. CK: before transplantation, FMT: fecal microbiota transplantation group (young rats, transplanted with microbiota from aged rats for 2 months), NFMT: non-fecal microbiota transplantation group (young rats, given PBS by gavage for 2 months). (**B**) Weighted UniFrac NMDS. F1-F5: FMT1-5, NF1-5: NFMT1-5. (**C**) UPGMA clustering analysis based on Weighted UniFrac distance. (**D**) Relative abundance of microbiota at phylum in each sample. (**E**) Phyla with significant differences between NFMT and FMT rats. (**F**) The ratio of F/B. (**G**) Heatmap at genus level; red represents an increase, and blue represents a decrease compared with NFMT rats. (**H**) Genera with significant differences between NFMT and FMT rats; * *P* < 0.05, ** *P* < 0.01 versus NFMT rats; ^#^ *P* < 0.05, ^##^ *P* < 0.01 versus CK.

The diversity of gut microbiota in the FMT group was lower than in the non-fecal microbiota transplantation (NFMT) group, although the difference was not statistically significant (Figure 5A). NMDS analysis showed that the gut microbiota of the FMT rats was significantly different from the NFMT rats (*P* < 0.05, Figure 5B). UPGMA cluster analysis also showed that the microbiota in the control NFMT group was closer to the microbiota before transplantation, while the microbiota in the FMT group was significantly different from the NFMT group, and was closer to aged rats (Figure 5C).

Microbial community analysis also showed that FMT changed the gut microbiota of the young recipient rats. At the phylum level, the relative abundance of *Bacteroidetes* was lower in FMT rats than in NFMT rats, and the difference was significant (*P* < 0.01). In contrast, the abundance of *Firmicutes* was higher, although the difference was not statistically significant (Figure 5D–5E). Compared with NFMT group, the ratio of F/B in FMT group increased (*P* < 0.05, Figure 5F). The relative abundance of *Actinobacteria* increased in FMT group, although the difference was not statistically significant. The relative abundance of *Spirochaetes* did not change in FMT group (Figure 5E). At the genus level, FMT rats had lower levels of *Prevotella*, *Bacteroide*, *Parabacteroides,* and higher levels of *Sutterella* (Figure 5G–5H)*.*

### FMT of aged microbiota from aged rats impairs cognitive behavior in young rats

**Figure 6:**
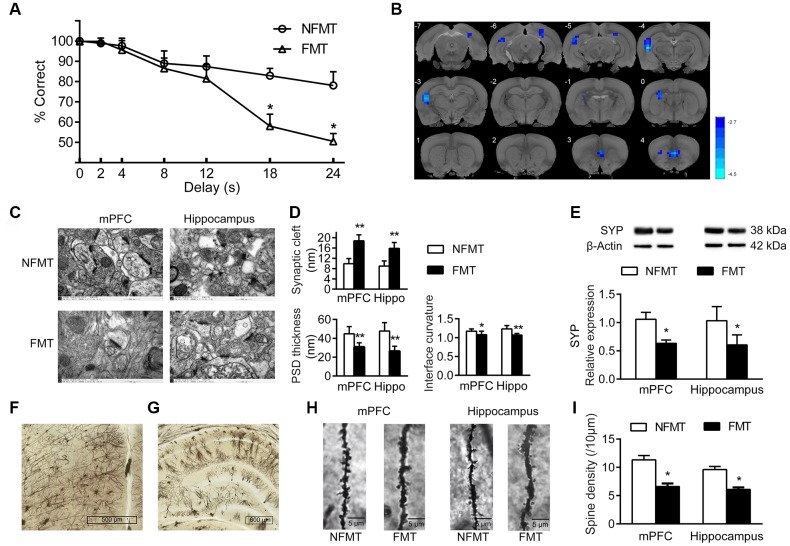
**Effect of FMT on cognition in young recipient rats.** (**A**) Effect of FMT on cognitive behavior by DMTP task; n = 12. (**B**) Images of brain slices showing regions with lower ReHo in FMT group compared with NFMT group (n = 20, voxel level < 0.005, cluster level < 0.05 GRF corrected, and clusters > 50 voxels). Blue denotes decreased ReHo; the color bars indicated the T value between two groups. (**C**) Synaptic structures of mPFC and hippocampus in NFMT and FMT rats. n = 3. (**D**) Histograms of synaptic structure parameters. n = 3. (**E)** Expression of synaptophysin in mPFC and hippocampus. (**F**–**I**) Golgi staining in NFMT and FMT rats (n = 3). Representative Golgi staining images of mPFC (**F**) and hippocampus (**G**) demonstrating impregnation of neurons. (**H**) Representative images of dendritic spines. (**I**) Histogram of dendritic spines densities; * *P* < 0.05 and ** *P* < 0.01 versus the NFMT group.

The cognitive behavior of NFMT and FMT rats was evaluated by DMTP task. The correct rate of lever-pressing under different delays is shown in Figure 6A. At the shortest delay, both NFMT and FMT rats performed well. However, at longer intervals (18 s and 24 s), the accuracy of lever-pressing in FMT rats was significantly lower than in the NFMT group (*P* < 0.05, Figure 6A), suggesting that the cognition of the young recipient rats was impaired by the FMT of aged microbiota.

### FMT of aged microbiota decreases brain activity in young rats

Functional Magnetic Resonance Imaging (fMRI) is one of the best tools for studying functions of the brain. As with conventional fMRI, resting state fMRI (rs-fMRI) typically measures BOLD signals reflecting the brain hemodynamics. In contrast with conventional fMRI, rs-fMRI is a task-free technique that monitors the intrinsic and fundamental activities of the brain neural circuits [10]. ReHo is a method to assess the rs-fMRI data, which is used to measure the BOLD synchrony of a given voxel to those of its nearest surrounding voxels, thus measuring the consistency of neuronal activity in local brain regions in time series [11]. A higher ReHo value reflects a higher local BOLD synchrony, representing an increase in neuron activity in the particular brain area. In contrast, a lower ReHo indicates a decrease in neuron activity in the particular brain area. Our results showed that the FMT rats possessed a lower ReHo in mPFC and hippocampus compared with the NFMT rats, indicating that FMT decreased the functional activities in these two brain areas in the young recipient rats (Figure 6B).

### FMT of aged microbiota changes synaptic structures and dendritic spines in young rats

To evaluate the alterations of neuronal structures induced by FMT, we analyzed the effect of FMT on synaptic structures, synaptophysin expression, and density of dendritic spines in mPFC and hippocampus. Compared with NFMT rats, the synaptic structures of FMT rats were significantly changed and similar to the structural characteristics of aged rats, including widened synaptic cleft (mPFC: 18.82 ± 2.30 vs 9.92 ± 1.97, *P* < 0.01; hippocampus: 15.87 ± 2.30 vs 8.99 ± 2.00, *P* < 0.01), decrease in the thickness of post-synaptic density (mPFC: 30.79 ± 4.49 vs 44.84 ± 7.58, *P* < 0.01; hippocampus: 26.41 ± 5.21 vs 47.76 ± 8.85, *P* < 0.01), as well as a decrease in the curvature of synaptic interface (mPFC: 1.08 ± 0.09 vs 1.17 ± 0.06, *P* < 0.05; hippocampus: 1.07 ± 0.04 vs 1.23 ± 0.09, *P* < 0.01, Figure 6C, 6D). In addition, the expression of synaptophysin in mPFC and hippocampus of FMT rats was decreased compared with NFMT rats (*P* < 0.05, Figure 6E). In mPFC and hippocampus, the dendritic spines of FMT rats decreased significantly compared with NFMT rats (*P* < 0.05, Figure 6F–6I).

### FMT of aged microbiota changes expression of NR1, BDNF, and AGE/RAGE

**Figure 7:**
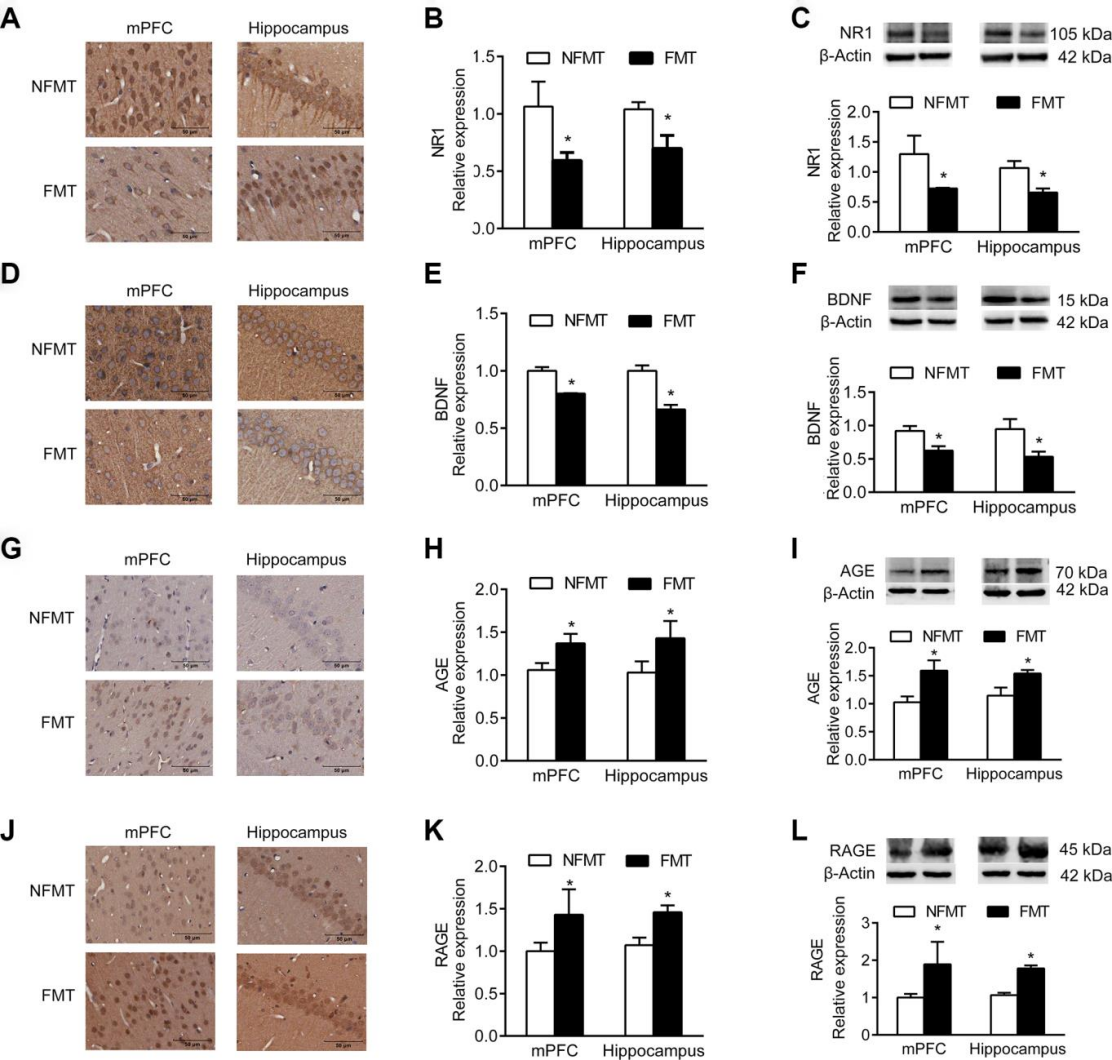
**Effect of FMT on expression of molecular markers. n = 3.** (**A**–**C**) FMT decreases expression of NR1 by IHC (**A**, **B**) and western blotting (**C**). (**D**–**F**) FMT decreases expression of BDNF by IHC (**D**, **E**) and western blotting (**F**). (**G**–**I**) FMT increases expression of AGE by IHC (**G**–**H**) and western blotting (**I**). (**J**–**L**) FMT increases expression of RAGE by IHC (**J**–**K**) and western blotting (**L**). Error bars represent SEM; * *P* < 0.05 versus the NFMT group.

NR1, an essential subunit of the NMDA receptor, and BDNF are important signaling proteins involved in neuronal proliferation, differentiation, synaptic plasticity, and survival. Immunoblotting and IHC analyses showed that the expression of NR1 and BDNF in mPFC and hippocampus of FMT rats was significantly decreased, and similar to that in aged rats, compared with NFMT rats (*P* < 0.05, Figure 7A–7F).

Advanced glycation end products (AGEs) and receptor for AGEs (RAGE) accumulate in neurons during normal aging, and have been used as markers of normal aging [12, 13]. Immunoblotting and IHC showed that the expression of AGE and RAGE in mPFC and hippocampus of FMT rats significantly increased compared with NFMT rats, having the characteristics of aged rats (*P* < 0.05, Figure 7G–7L).

### FMT of aged microbiota increases inflammatory cytokine levels in young rats

**Figure 8:**
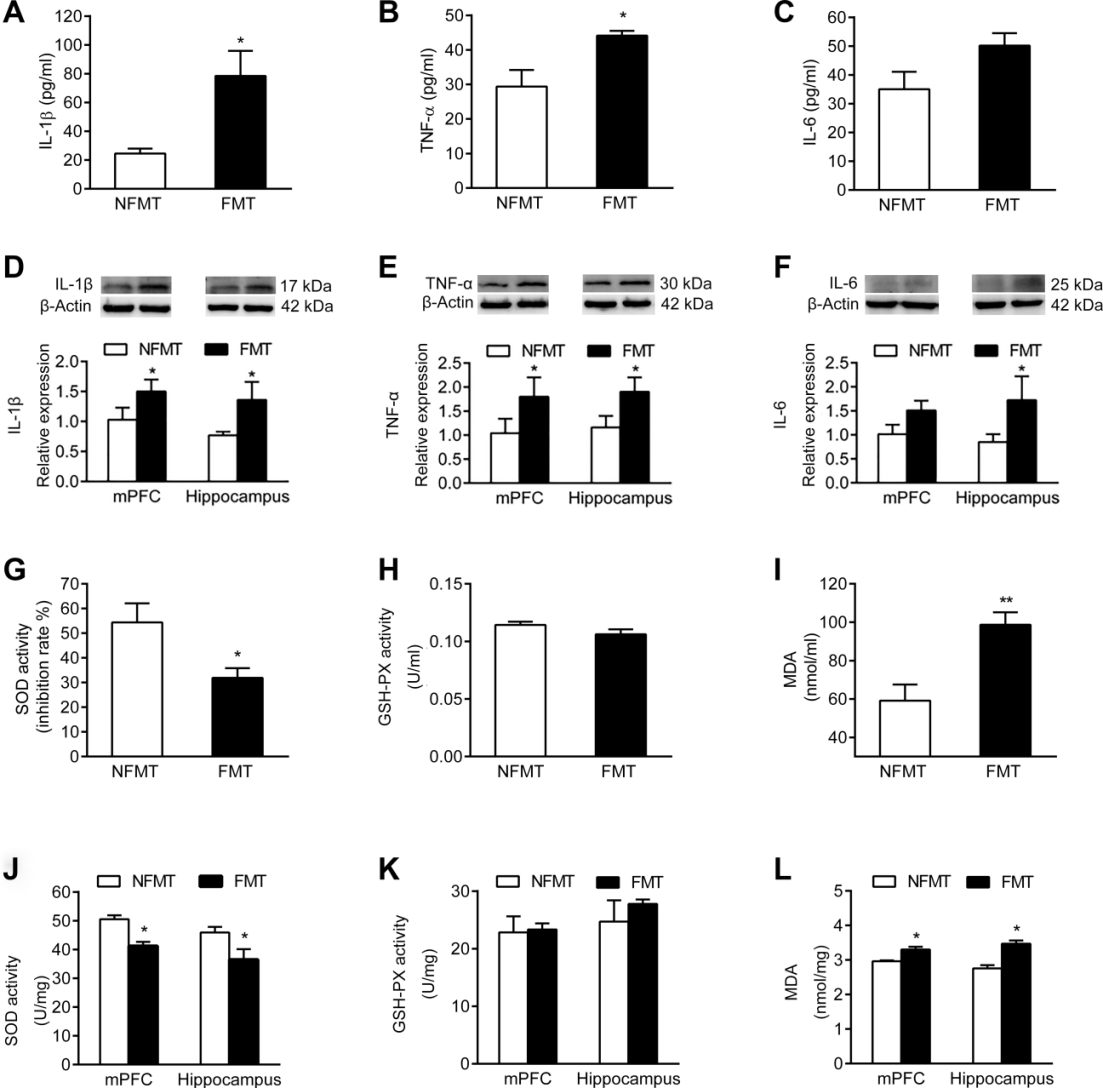
**Effect of FMT on inflammation and oxidative stress.** n = 6. FMT increases serum levels of IL-1β (**A**) TNF-α (**B**) and IL-6 (**C**). FMT increases expression of IL-1β (**D**) TNF-α (**E**) and IL-6 (**F**) in mPFC and hippocampus. Effect of FMT on SOD activity (**G**) GSH-PX activity (**H**) and MDA content (**I**) in serum. Effect of FMT on SOD activity (**J**) GSH-PX activity (**K**) and MDA content (**L**) in mPFC and hippocampus. Error bars represent SEM; * *P* < 0.05 and ** *P* < 0.01 versus the NFMT group.

Having shown that the levels of the inflammatory cytokines IL-1β, TNF-α and IL-6 increase in serum and brain of aged rats (Figure 3A–3F), we asked whether FMT of aged microbiota increases the inflammatory cytokine levels in young rats. Both serum and brain levels of IL-1β, TNF-α and IL-6 were significantly increased in FMT rats compared with NFMT rats (*P* < 0.05, Figure 8A–8F). These results suggest that inflammation might be one of the mechanisms by which gut microbiota contributes to the cognitive decline in normal aging.

### FMT of aged microbiota increases oxidative stress in young rats

Since our results showed that the SOD activity in serum and brain declined, the MDA content increased, and the activity of GSH-PX did not change in aged rats (Figure 3G–3L), we tested whether the FMT of aged microbiota will cause similar changes in young rats. In serum, the SOD activity of FMT rats declined, and the MDA content increased compared with NFMT rats (*P* < 0.05, Figure 8G, 8I). The SOD activity also declined and the MDA content increased in mPFC and hippocampus of FMT rats compared with NFMT rats (*P* < 0.05, Figure 8J, 8L). The activity of GSH-PX did not change in serum and brain (Figure 8H, 8K). These results suggest that oxidative stress might represent another mechanism contributing to the cognitive decline in normal aging.

## DISCUSSION

With advancing age, cognitive dysfunction becomes more common, especially fluid intelligence including processing speed, working memory, and long-term memory [14]. Among these, the working memory has been found to be particularly sensitive. To assess the working memory deficits, delayed response tasks have been particularly valuable experimental models. The experimental model named “delayed matching to position” (DMTP) was originally designed as an analogue of the primate test “delayed matching to sample” [15]. Different from the maze task used in most cognitive experiments, DMTP allows a distinction between mnemonic impairments and non-specific impairments (such as impaired retention of the matching rule, diminished attentiveness to the task and general motivational, motor, or sensory impairments). In the present study, we employed the DMTP task to assess the working memory in aged rats. In this test, aged rats performed well at the shortest delay intervals, and showed no detectable deficit in learning the task contingencies, indicating that non-mnemonic cognitive functions, such as attention and motivation, were unimpaired. However, as the delay lengthened, aged rats showed progressive impairments, suggesting an explicit impairment in the retention of information in the working memory.

Comparing the gut microbiota of aged and young rats revealed that the gut microbiota of the aged rats was significantly different from the young rats, especially in the dominant bacteria *Bacteroidetes* and *Firmicutes*. It has been widely accepted that the gut microbiota composition changes through aging [16]. Previous studies have shown a decreased ratio of *Firmicutes* to *Bacteroides* (F/B) [17], increase in the facultative anaerobes *Staphylococcus* and *Bacillus*, increase in *E. coli* [5], and a decrease in *Bifidobacteria* in the elderly [18]. However, the age-related variations in gut microbiota have not been consistent. For example, different results have been found in the numbers of *Bacteroides* between young and elderly subjects [18, 19]. Another pronounced change is the reduction in the microbiota diversity with increasing age [6]; microbiota diversity is a desirable trait that confers resistance to environmental stressors [20]. The age-related loss of diversity decreases resistance to colonization with pathogens, and may account for the higher rates of *C. difficile* colitis and other infections in the elderly. Furthermore, the age-related perturbations in the gut microbiota initiate age-related pathological states, such as chronic inflammation, cognitive decline, and frailty. In most cases, the gut microbiota of older adults represents a pro-inflammatory phenotype [6].

Recent animal studies have indicated that the gut microbiota affects learning and memory [7]. For example, one study showed an absence of non-spatial and working memory in GF (Germ Free) mice compared with SPF (Specified Pathogen Free) mice colonized with intact gut microbiota [8]. Another report showed that a combination of antibiotics (ampicillin, bacilli, meropenem, neomycin, and vancomycin) resulted in an impaired recognition memory of novel objects in SPF mice [9]. In addition, mice exposed to water avoidance stress (an aversive stimulus) after infection with *C. rodentium*, had an impaired memory, but this memory dysfunction was prevented by daily treatment with probiotics [8]. However, since until now, the published reports were based on special animal models, such as GF mice, or on artificial interventions that change the gut microbiota, such as pathogenic bacteria infection, probiotics, or antibiotics, and did not consider the aging process or aging biological characteristics, our understanding of the association between the gut microbiota and cognitive function under normal aging process has been limited.

In order to explore the effect of gut microbiota on cognitive dysfunction in aging, we employed the FMT, a direct method to change the recipients’ gut microbiota. We replaced the young recipient rats’ gut microbiota with the microbiota obtained from aged rats. The FMT reshaped the gut microbiota in young recipient rats so that it resembled the microbiota of aged rats; these changes included the decreased diversity as well as the changed proportion of *Bacteroidetes* and *Firmicutes*. In addition, the young rats that received the aged microbiota by FMT had significant cognitive behavioral impairments, suggesting that the change in gut microbiota contributed to the cognitive decline in aged rats.

Cognitive aging is reflected not only by behavior, but also by the changes of brain structures and functions. Rs-fMRI reflects the changes of spontaneous neural activity in the resting-state. ReHo measures the regional homogeneity, i.e., similarity. It assumes that within a functional cluster, the hemodynamic characteristics of every voxel are similar to its neighbors, and that such similarity could be changed or modulated by different conditions [11]. Several studies have investigated the regional homogeneity in Alzheimer's disease (AD) and mild cognitive impairment (MCI) patients, in which the patients showed significant decreased ReHo values in the medial prefrontal cortex (mPFC), the bilateral posterior cingulate gyrus/precuneus (PCC/PCu), and the left inferior parietal lobule (IPL). In addition, the altered ReHo index correlated with the clinical variables in MCI and AD patients, suggesting that the spontaneous activity pattern in the resting state could be used as a marker for clinical diagnoses of MCI/AD [21, 22]. Furthermore, a previous study indicated that normal aging decreases the regional homogeneity in extensive brain regions [23]. In agreement with these studies, our data showed that aged rats had significantly decreased ReHo values in almost the entire brain. Importantly, young rats transplanted with the aged microbiota had decreased ReHo values, especially in mPFC and hippocampus, which are the two crucial areas for cognitive functions. Comparing behavior and ReHo values of aged and young recipient rats revealed that both groups had similar behavioral responses with obvious errors in longer delayed intervals in DMTP test; however, significant differences in ReHo values were observed between these two groups. In aged rats, the ReHo values were decreased in almost entire brain, while the young recipient rats showed local changes just in mPFC and hippocampus, indicating that FMT of aged microbiota may prefer to alter the cognition relative structures and processes, rather than general brain functions of the young recipient rats. Obviously, the ReHo values showed here were logical, reasonable, and acceptable results. In addition to the functional alterations, FMT induced changes of synaptic structures in mPFC and hippocampus, down-regulated expression of NR1, synaptophysin, and BDNF, and up-regulated expression of AGEs and RAGE. These structural and molecular changes were consistent with the behavioral changes and rs-fMRI results, demonstrating that the alterations induced by FMT of aged microbiota in young recipient rats are similar to aged rats.

Aging is characterized by a chronic, low-grade inflammation, also referred to as inflammaging [24]. The pro-inflammatory cytokines IL-6, TNFα, and IL-1 contribute to inflammaging in healthy elderly individuals, as well as in many age-related diseases [25, 26]. A great deal of evidence supports a link between systemic low-grade inflammation and cognitive decline in both normal and pathological aging [27–29]. Our results showed that aged rats had increased serum and brain levels of IL-1β, TNF-α, and IL-6 compared to young rats. The underlying cause of the age-associated inflammation is still debated. A recent study demonstrated that age-related gut microbial dysbiosis led to increased gut permeability and translocation of bacterial components, further fueling inflammation in the context of the aged host [30]. To decrease leaky gut and inflammation can improve physical and cognitive functions [31]. In our study, FMT of aged microbiota increased the brain and serum levels of IL-1β, TNF-α and IL-6 in the young rats. We speculated that FMT of aged microbiota increased gut leakage and systemic inflammation, resulted in cognitive decline. These results suggested that inflammation might be one of the mechanisms by which shifts in gut microbiota contribute to the age-related cognitive decline.

Oxidative stress is a common mechanism in the pathogenesis of various of diseases, including cognitive decline [32]. With increasing age, free radicals tend to increase, while antioxidant defense mechanisms decrease. Malondialdehyde (MDA) is a product of lipid peroxidation, which is an important index of oxidative damage caused by oxidative stress [33]; its expression is increased in the temporal cortex of the elderly [34]. At the same time, the body has very effective antioxidant defense mechanisms, including the antioxidant enzymes superoxide dismutase (SOD), catalase, glutathione peroxidase (GSH-PX), and glutathione reductase. Several studies have suggested that oxidative stress might be an important mechanism for intestinal micro-ecology to exert its biological effects. It was found that beta-N-methylamino-L-alanine, a neurotoxic substance produced by cyanobacteria in intestinal flora, can cause multiple sclerosis, AD, and PD by activating AMPA receptors or inducing oxidative stress to degrade glutathione [35]. Moreover, the intestinal *Lactobacillus* has an antioxidant capacity, which can remove reactive oxygen species (ROS) in the intestinal tract, and keep ROS in a relatively stable state [36]. Our results showed that FMT of aged microbiota in young rats increased the MDA content and decreased the SOD activity in serum and brain, suggesting that oxidative stress might represent another mechanism by which gut microbiota contributes to the age-related cognitive decline.

Together, our results indicate that the age-related changes in gut microbiota play an important role in cognitive impairment of the aging brain, suggesting that restoring the microbiota homeostasis in the elderly people might improve their physical and mental health. To our knowledge, this study provides the first direct evidence of the contribution of gut microbiota to the cognitive decline in normal aging process.

## MATERIALS AND METHODS

### Rats

Young (~ 3 months) and aged (20 ~ 24 months) male SD rats were obtained from Chengdu Dashuo Laboratory Animal Co., Ltd (Chengdu, China). The rats were specific-pathogen-free (SPF) grade animals, and were fed in a barrier SPF environment under 12 h light / dark cycle. Two rats were housed in each cage. All rats were fed autoclaved chow and sterilized water ad libitum. All protocols were reviewed and approved by the Ethics Committee of Shanxi Medical University.

### Delayed matching to position (DMTP) task

The tasks were conducted in well-controlled rat two-lever operant chambers (Med Associates, USA). The task consisted of a sample phase and a choice phase, separated by a variable delay. The rats were trained and tested as described previously [37]. During the initial sessions, there was 0-delay between the sample and choice phases. Once a rat reached >80% correct over 3 days, sets of delays were introduced (Set 1: 0, 1, 2, 3, 4, 5, 6 s; Set 2: 0, 1, 2, 4, 8, 12, 16 s; Set 3: 0, 2, 4, 8, 12, 18, 24 s). Upon establishing >80% correct over 3 days in Set 1, rats were progressed to Set 2. Each rat was tested for 5 consecutive sessions in Set 3.

### Fecal sample collection and 16S rRNA gene sequencing

The fecal samples were collected directly from the anus of each rat. The total genomic DNA was extracted from each sample by the QIAamp DNA stool mini kit (Qiagen, Hilden, Germany). The extracted DNA was subjected to PCR to amplify the variable V3-V4 region of the 16S locus using the universal bacterial primers 338F/806R on the Illumina MiSeq platform, as described [38].

For bioinformatics analysis, overlapping paired-end reads form original DNA fragments were filtered to obtain clean reads by using QIIME (version 1.17). Then, to get tags, clean reads were paired using FLASH (version 1.2.11). The tags were clustered to Operational Taxonomic Unit (OTUs) by utilizing the software USEARCH (v7.0.1090). To describe the alpha diversity, the numbers of OTUs and Shannon diversity index were calculated using R package (v3.1.1). Nonmetric Multidimensional Scaling (NMDS) analysis based on weighted UniFrac distances was used to display the difference of OTU composition among samples using R package. UPGMA clustering analysis of weighed UniFrac distance was carried out Using QIIME and R package to evaluate the similarity among samples. Next, the OTU representative sequences were classified taxonomically using Ribosomal Database Project (RDP) Classifier v.2.2, using 0.8 confidence values as cutoff. Statistical analyses were conducted in R (v3.1.1).

### Resting-state functional magnetic resonance imaging

All rats were scanned using 7.0T Bruker Scanner MRI system (Pharmascan, Bruker, Switzerland). T2-weighted anatomical images were acquired first, using a T2_TurboRARE sequence with the following parameters: repetition time (TR) = 5,300 ms, echo time (TE) = 33 ms, number of averages = 2, field of view = 35 mm × 35 mm, slice thickness = 0.55 mm, slices = 50, matrix = 256 × 256, flip angle = 90°. Functional scans were acquired using a T2Star_FID_EPI sequence with the following parameters: TR = 2000 ms, TE = 20 ms, number of averages = 1, field of view = 35 mm × 35 mm, slice thickness = 0.55 mm, slices = 50, matrix = 64 × 64, flip angle = 90°, 600 Repetitions. All original Bruker images were converted to DICOM format with the Paravision.

Preprocessing of all acquired resting-state data and the ReHo analysis were carried out using Statistical Parametric Mapping (SPM8, http://www.fil.ion.ucl.ac.uk/spm/software/spm8) and Data Processing Assistant for Resting-State fMRI (DPARSF, http://www.restfmri.net/forum/DPARSF). The dataset was spatially normalized to the standard rat brain atlas [39]. As described previously [23], ReHo analysis was performed using the REST software (http://restfmri.net/forum/REST). Individual ReHo maps were generated by calculating Kendall’s coefficient of concordance of the time series of a given voxel with those of its nearest 26 neighboring voxels. Then, all ReHo maps were smoothed with an isotropic Gaussian kernel of 2 mm (full-width half-maximum). The two sample *t*-test was performed to determine the difference between two groups. Clusters that were significantly different were selected by setting *P* < 0.005 with a Gaussian random fields (GRF) correction and clusters more than 50 voxels.

### Fecal microbiota transplantation (FMT)

Before transplantation, the FMT rats received an antibiotic mixture consisting of ampicillin (180 mg/kg/d), vancomycin (72 mg/kg/d), metronidazole (90 mg/kg/d) and imipenem (90 mg/kg/d), twice daily (12 h interval) for three consecutive days [40]. Transplantation was conducted at day 4 (at least 24 h from the last antibiotic treatment). To prepare suspension of fecal bacteria for the transplantation, the fresh fecal contents of aged rats were surgically extracted, pooled, and diluted with PBS, as described [41]. The suspension was centrifuged at 800 rpm for 5 min. The supernatant was taken and given to the recipient rats by gavage immediately. The rats underwent FMT once a day for three days, then twice a week for two months.

### Golgi staining

According to the instruction of FD Rapid Golgi Stain Kit (FD Neuro Technologies, Inc. USA), the tissues were cut into 100 μm-thick slices and mounted on gelatin-coated slides. After staining, the sections were examined under a light microscope. All images were analyzed by Image J software. The dendritic Spine density was expressed as spine number per 10 μm dendritic length.

### Transmission electron microscopy

The synaptic ultrastructure features were observed under transmission electron microscope (JEM -100CX, JEOL, Japan), and gray type-I synapses were analyzed. The width of synaptic gap, the thickness of PSD, and the curvature of synaptic interface were quantitatively analyzed with Image J, as described [42].

### Western blotting

Equal amounts of protein were separated by SDS-PAGE and transferred to a PVDF membrane. After blocking with 5% BSA, immunoblots were incubated with the following specific primary antibodies: NMDAR1 (1:1000, ab68144, Abcam), BDNF (1:1000, ab216443, Abcam), Synaptophysin (1:20000, ab32127, Abcam), AGEs (1:500, ab23722, Abcam), RAGE (1:500, ab3611, Abcam), IL-1β (1:500, ab9722, Abcam), TNF-α (1:1000, ab6671, Abcam), IL-6 (1:1000, ab9324, Abcam) and β-actin (1:400, BM0627, Boster). After incubation with HRP-labeled secondary antibodies, the bands were visualized using the ECL detection kit. The protein bands were quantitatively analyzed by using Image Lab software 5.2.

### Immunohistochemistry

Paraffin-embedded tissues were cut into 4 μm-thick sections, and incubated with a primary antibody, followed by enzyme-labelled secondary antibody. The primary antibodies used were: NMDAR1 (1:100, ab68144, Abcam), BDNF (1:500, ab216443, Abcam), AGE (1:100, ab23722, Abcam), and RAGE (1:20, ab3611, Abcam). After incubation with a secondary antibody, the sections were visualized by using 3,3′-diaminobenzidine (DAB). Images were photographed and analyzed with Digital Pathology Solutions (Scanscope CS, Aperio, USA).

### Enzyme-linked immunosorbent assay (ELISA)

Serum was collected by centrifugation at 1000 g for 10 min at 4°C. IL-1β, TNF-α, and IL-6 serum levels were analyzed by commercially available ELISA kits (SHANGHAI WESTANG BIO-TECH).

### Analysis of oxidative stress level

Serum was collected as above, and the activities of SOD (ab65354, Abcam) and GSH-PX (ab102530, Abcam), and the level of MDA (ab118970, Abcam) were analyzed according to the manufacturer’s instructions.

The medial prefrontal cortex and hippocampus were homogenized, and the homogenates were centrifuged at 3000 rpm for 15 min. The supernatants were collected, and analyzed for the activities of SOD (A001; Nanjing Jiancheng Bioengineering Institute, Nanjing, China), GSH-PX (A005; Nanjing Jiancheng Bioengineering Institute, Nanjing, China), and the content of MDA (A003; Nanjing Jiancheng Bioengineering Institute, Nanjing, China).

### Statistical analysis

Data are presented as means ± SEMs. Statistical analysis was performed by using SPSS 13.0. Two-factor repeated-measures ANOVA was used to analyze the performance of DMTP Task. To compare the data between two groups, *t*-test was used; one-way ANOVA was performed to analyze data from three or more groups. Difference was considered significant if *P* < 0.05.

## References

[1] Partridge L. Evolutionary theories of ageing applied to long-lived organisms. Exp Gerontol. 2001; 36:641–50. 10.1016/S0531-5565(00)00232-1 11295505

[2] Thursby E, Juge N. Introduction to the human gut microbiota. Biochem J. 2017; 474:1823–36. 10.1042/BCJ20160510 28512250 PMC5433529

[3] Bercik P, Collins SM, Verdu EF. Microbes and the gut-brain axis. Neurogastroenterol Motil. 2012; 24:405–13. 10.1111/j.1365-2982.2012.01906.x 22404222

[4] den Besten G, van Eunen K, Groen AK, Venema K, Reijngoud DJ, Bakker BM. The role of short-chain fatty acids in the interplay between diet, gut microbiota, and host energy metabolism. J Lipid Res. 2013; 54:2325–40. 10.1194/jlr.R036012 23821742 PMC3735932

[5] Claesson MJ, Cusack S, O’Sullivan O, Greene-Diniz R, de Weerd H, Flannery E, Marchesi JR, Falush D, Dinan T, Fitzgerald G, Stanton C, van Sinderen D, O’Connor M, et al. Composition, variability, and temporal stability of the intestinal microbiota of the elderly. Proc Natl Acad Sci USA. 2011 (Suppl 1); 108:4586–91. 10.1073/pnas.1000097107 20571116 PMC3063589

[6] Claesson MJ, Jeffery IB, Conde S, Power SE, O’Connor EM, Cusack S, Harris HM, Coakley M, Lakshminarayanan B, O’Sullivan O, Fitzgerald GF, Deane J, O’Connor M, et al. Gut microbiota composition correlates with diet and health in the elderly. Nature. 2012; 488:178–84. 10.1038/nature11319 22797518

[7] Schroeder BO, Bäckhed F. Signals from the gut microbiota to distant organs in physiology and disease. Nat Med. 2016; 22:1079–89. 10.1038/nm.4185 27711063

[8] Gareau MG, Wine E, Rodrigues DM, Cho JH, Whary MT, Philpott DJ, Macqueen G, Sherman PM. Bacterial infection causes stress-induced memory dysfunction in mice. Gut. 2011; 60:307–17. 10.1136/gut.2009.202515 20966022

[9] Fröhlich EE, Farzi A, Mayerhofer R, Reichmann F, Jačan A, Wagner B, Zinser E, Bordag N, Magnes C, Fröhlich E, Kashofer K, Gorkiewicz G, Holzer P. Cognitive impairment by antibiotic-induced gut dysbiosis: analysis of gut microbiota-brain communication. Brain Behav Immun. 2016; 56:140–55. 10.1016/j.bbi.2016.02.020 26923630 PMC5014122

[10] Akbari E, Asemi Z, Daneshvar Kakhaki R, Bahmani F, Kouchaki E, Tamtaji OR, Hamidi GA, Salami M. Effect of Probiotic Supplementation on Cognitive Function and Metabolic Status in Alzheimer’s Disease: A Randomized, Double-Blind and Controlled Trial. Front Aging Neurosci. 2016; 8:256. 10.3389/fnagi.2016.00256 27891089 PMC5105117

[11] Zang Y, Jiang T, Lu Y, He Y, Tian L. Regional homogeneity approach to fMRI data analysis. Neuroimage. 2004; 22:394–400. 10.1016/j.neuroimage.2003.12.030 15110032

[12] Ramasamy R, Vannucci SJ, Yan SS, Herold K, Yan SF, Schmidt AM. Advanced glycation end products and RAGE: a common thread in aging, diabetes, neurodegeneration, and inflammation. Glycobiology. 2005; 15:16R–28R. 10.1093/glycob/cwi053 15764591

[13] Sato Y, Kondo T, Ohshima T. Estimation of age of human cadavers by immunohistochemical assessment of advanced glycation end products in the hippocampus. Histopathology. 2001; 38:217–20. 10.1046/j.1365-2559.2001.01059.x 11260301

[14] Park DC, Polk TA, Hebrank AC, Jenkins LJ. Age differences in default mode activity on easy and difficult spatial judgment tasks. Front Hum Neurosci. 2010; 3:75. 10.3389/neuro.09.075.2009 20126437 PMC2814559

[15] Dunnett SB, Evenden JL, Iversen SD. Delay-dependent short-term memory deficits in aged rats. Psychopharmacology (Berl). 1988; 96:174–80. 10.1007/BF00177557 3148143

[16] Sharon G, Sampson TR, Geschwind DH, Mazmanian SK. The Central Nervous System and the Gut Microbiome. Cell. 2016; 167:915–32. 10.1016/j.cell.2016.10.027 27814521 PMC5127403

[17] Mariat D, Firmesse O, Levenez F, Guimarăes V, Sokol H, Doré J, Corthier G, Furet JP. The Firmicutes/Bacteroidetes ratio of the human microbiota changes with age. BMC Microbiol. 2009; 9:123. 10.1186/1471-2180-9-123 19508720 PMC2702274

[18] Hopkins MJ, Macfarlane GT. Changes in predominant bacterial populations in human faeces with age and with Clostridium difficile infection. J Med Microbiol. 2002; 51:448–54. 10.1099/0022-1317-51-5-448 11990498

[19] Woodmansey EJ, McMurdo ME, Macfarlane GT, Macfarlane S. Comparison of compositions and metabolic activities of fecal microbiotas in young adults and in antibiotic-treated and non-antibiotic-treated elderly subjects. Appl Environ Microbiol. 2004; 70:6113–22. 10.1128/AEM.70.10.6113-6122.2004 15466557 PMC522128

[20] Yachi S, Loreau M. Biodiversity and ecosystem productivity in a fluctuating environment: the insurance hypothesis. Proc Natl Acad Sci USA. 1999; 96:1463–68. 10.1073/pnas.96.4.1463 9990046 PMC15485

[21] Zhang Z, Liu Y, Jiang T, Zhou B, An N, Dai H, Wang P, Niu Y, Wang L, Zhang X. Altered spontaneous activity in Alzheimer’s disease and mild cognitive impairment revealed by Regional Homogeneity. Neuroimage. 2012; 59:1429–40. 10.1016/j.neuroimage.2011.08.049 21907292

[22] Bernier M, Croteau E, Castellano CA, Cunnane SC, Whittingstall K. Spatial distribution of resting-state BOLD regional homogeneity as a predictor of brain glucose uptake: A study in healthy aging. Neuroimage. 2017; 150:14–22. 10.1016/j.neuroimage.2017.01.055 28130193

[23] Wu T, Zang Y, Wang L, Long X, Li K, Chan P. Normal aging decreases regional homogeneity of the motor areas in the resting state. Neurosci Lett. 2007; 423:189–93. 10.1016/j.neulet.2007.06.057 17709202

[24] Atienza M, Ziontz J, Cantero JL. Low-grade inflammation in the relationship between sleep disruption, dysfunctional adiposity, and cognitive decline in aging. Sleep Med Rev. 2018; 42:171–83. 10.1016/j.smrv.2018.08.002 30241997

[25] Franceschi C, Bonafè M, Valensin S, Olivieri F, De Luca M, Ottaviani E, De Benedictis G. Inflamm-aging. An evolutionary perspective on immunosenescence. Ann N Y Acad Sci. 2000; 908:244–54. 10.1111/j.1749-6632.2000.tb06651.x 10911963

[26] Franceschi C, Campisi J. Chronic inflammation (inflammaging) and its potential contribution to age-associated diseases. J Gerontol A Biol Sci Med Sci. 2014 (Suppl 1); 69:S4–9. 10.1093/gerona/glu057 24833586

[27] Mooijaart SP, Sattar N, Trompet S, Lucke J, Stott DJ, Ford I, Jukema JW, Westendorp RG, de Craen AJ, Group PS, and PROSPER Study Group. Circulating interleukin-6 concentration and cognitive decline in old age: the PROSPER study. J Intern Med. 2013; 274:77–85. 10.1111/joim.12052 23414490

[28] Schram MT, Euser SM, de Craen AJ, Witteman JC, Frölich M, Hofman A, Jolles J, Breteler MM, Westendorp RG. Systemic markers of inflammation and cognitive decline in old age. J Am Geriatr Soc. 2007; 55:708–16. 10.1111/j.1532-5415.2007.01159.x 17493190

[29] Chang R, Yee KL, Sumbria RK. Tumor necrosis factor α Inhibition for Alzheimer’s Disease. J Cent Nerv Syst Dis. 2017; 9:1179573517709278. 10.1177/1179573517709278 28579870 PMC5436834

[30] Thevaranjan N, Puchta A, Schulz C, Naidoo A, Szamosi JC, Verschoor CP, Loukov D, Schenck LP, Jury J, Foley KP, Schertzer JD, Larché MJ, Davidson DJ, et al. Age-Associated Microbial Dysbiosis Promotes Intestinal Permeability, Systemic Inflammation, and Macrophage Dysfunction. Cell Host Microbe. 2017; 21:455–466.e4. 10.1016/j.chom.2017.03.002 28407483 PMC5392495

[31] Wang S, Ahmadi S, Nagpal R, Jain S, Mishra SP, Kavanagh K, Zhu X, Wang Z, McClain DA, Kritchevsky SB, Kitzman DW, Yadav H. Lipoteichoic acid from the cell wall of a heat killed Lactobacillus paracasei D3-5 ameliorates aging-related leaky gut, inflammation and improves physical and cognitive functions: from C. elegans to mice. Geroscience. 2020; 42:333–52. 10.1007/s11357-019-00137-4 31814084 PMC7031475

[32] Bishop NA, Lu T, Yankner BA. Neural mechanisms of ageing and cognitive decline. Nature. 2010; 464:529–35. 10.1038/nature08983 20336135 PMC2927852

[33] Zhong SZ, Ge QH, Qu R, Li Q, Ma SP. Paeonol attenuates neurotoxicity and ameliorates cognitive impairment induced by d-galactose in ICR mice. J Neurol Sci. 2009; 277:58–64. 10.1016/j.jns.2008.10.008 19007942

[34] Dei R, Takeda A, Niwa H, Li M, Nakagomi Y, Watanabe M, Inagaki T, Washimi Y, Yasuda Y, Horie K, Miyata T, Sobue G. Lipid peroxidation and advanced glycation end products in the brain in normal aging and in Alzheimer’s disease. Acta Neuropathol. 2002; 104:113–22. 10.1007/s00401-002-0523-y 12111353

[35] Vyas KJ, Weiss JH. BMAA—an unusual cyanobacterial neurotoxin. Amyotroph Lateral Scler. 2009 (Suppl 2); 10:50–55. 10.3109/17482960903268742 19929732

[36] Hathout AS, Mohamed SR, El-Nekeety AA, Hassan NS, Aly SE, Abdel-Wahhab MA. Ability of Lactobacillus casei and Lactobacillus reuteri to protect against oxidative stress in rats fed aflatoxins-contaminated diet. Toxicon. 2011; 58:179–86. 10.1016/j.toxicon.2011.05.015 21658402

[37] Sloan HL, Good M, Dunnett SB. Double dissociation between hippocampal and prefrontal lesions on an operant delayed matching task and a water maze reference memory task. Behav Brain Res. 2006; 171:116–26. 10.1016/j.bbr.2006.03.030 16677723

[38] Fadrosh DW, Ma B, Gajer P, Sengamalay N, Ott S, Brotman RM, Ravel J. An improved dual-indexing approach for multiplexed 16S rRNA gene sequencing on the Illumina MiSeq platform. Microbiome. 2014; 2:6. 10.1186/2049-2618-2-6 24558975 PMC3940169

[39] Paxinos G, Watson C. The rat brain in stereotaxic coordinates. Amsterdam, Boston: Academic Press; 2005.

[40] Mell B, Jala VR, Mathew AV, Byun J, Waghulde H, Zhang Y, Haribabu B, Vijay-Kumar M, Pennathur S, Joe B. Evidence for a link between gut microbiota and hypertension in the Dahl rat. Physiol Genomics. 2015; 47:187–97. 10.1152/physiolgenomics.00136.2014 25829393 PMC4451389

[41] Manichanh C, Reeder J, Gibert P, Varela E, Llopis M, Antolin M, Guigo R, Knight R, Guarner F. Reshaping the gut microbiome with bacterial transplantation and antibiotic intake. Genome Res. 2010; 20:1411–19. 10.1101/gr.107987.110 20736229 PMC2945190

[42] Carter AR, Chen C, Schwartz PM, Segal RA. Brain-derived neurotrophic factor modulates cerebellar plasticity and synaptic ultrastructure. J Neurosci. 2002; 22:1316–27. 10.1523/JNEUROSCI.22-04-01316.2002 11850459 PMC6757568

